# A useful case of ultrasound-guided axillary lymph node aspiration in a breast cancer patient with improved needle visibility

**DOI:** 10.1016/j.radcr.2021.07.069

**Published:** 2021-08-26

**Authors:** Tomoyuki Fujioka, Mio Mori, Yuka Yashima, Emi Yamaga, Jun Oyama, Kota Yokoyama, Kazunori Kubota, Goshi Oda, Tsuyoshi Nakagawa, Iichiroh Onishi, Ukihide Tateishi

**Affiliations:** a Department of Diagnostic Radiology, Tokyo Medical and Dental University, 1-5-45, Yushima, Bunkyo-ku Tokyo, 113-8519 Japan; b Department of Radiology, Dokkyo Medical University Saitama Medical Center, 2-1-50, Minamikoshigaya, Koshigaya, Saitama, 343-8555 Japan; c Department of Surgery, Breast Surgery, Tokyo Medical and Dental University, 1-5-45, Yushima, Bunkyo-ku Tokyo, 113-8519 Japan; d Department of Diagnostic Pathology, Tokyo Medical and Dental University, 1-5-45, Yushima, Bunkyo-ku Tokyo, 113-8519 Japan

**Keywords:** Ultrasound, Fine-needle aspiration, Axillary lymph node, Breast cancer, Matrix linear probe

## Abstract

Ultrasound-guided, lymph node, fine-needle aspiration cytology is important in diagnosing axillary lymph node metastasis in breast cancer. However, poor needle visibility can render the procedure difficult. We describe a case in which state-of-the-art enhancement techniques using matrix linear probes can provide better needle visibility and improve the certainty and efficiency of the examination.

## Introduction

Ultrasound (US)-guided, axillary lymph node, fine-needle aspiration cytology (FNAC) is important for the determination of the treatment strategy for breast cancer [1]. However, the technique is difficult to perform owing to the poor needle visibility [2,3].

**Fig. 1:**
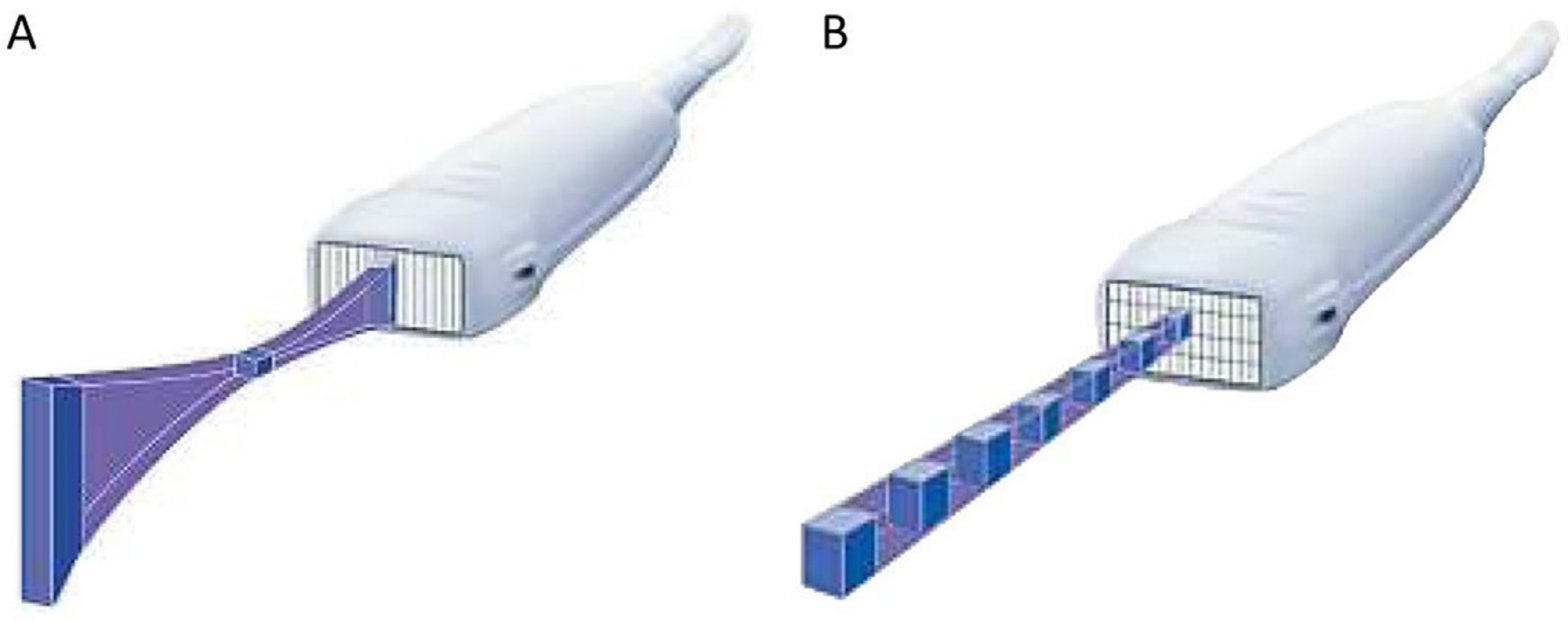
Conventional linear and matrix linear probes. Conventional linear probe (A) and matrix linear probe (B). Multiple elements are arranged in the thickness direction of the matrix probe.

**Fig. 2:**
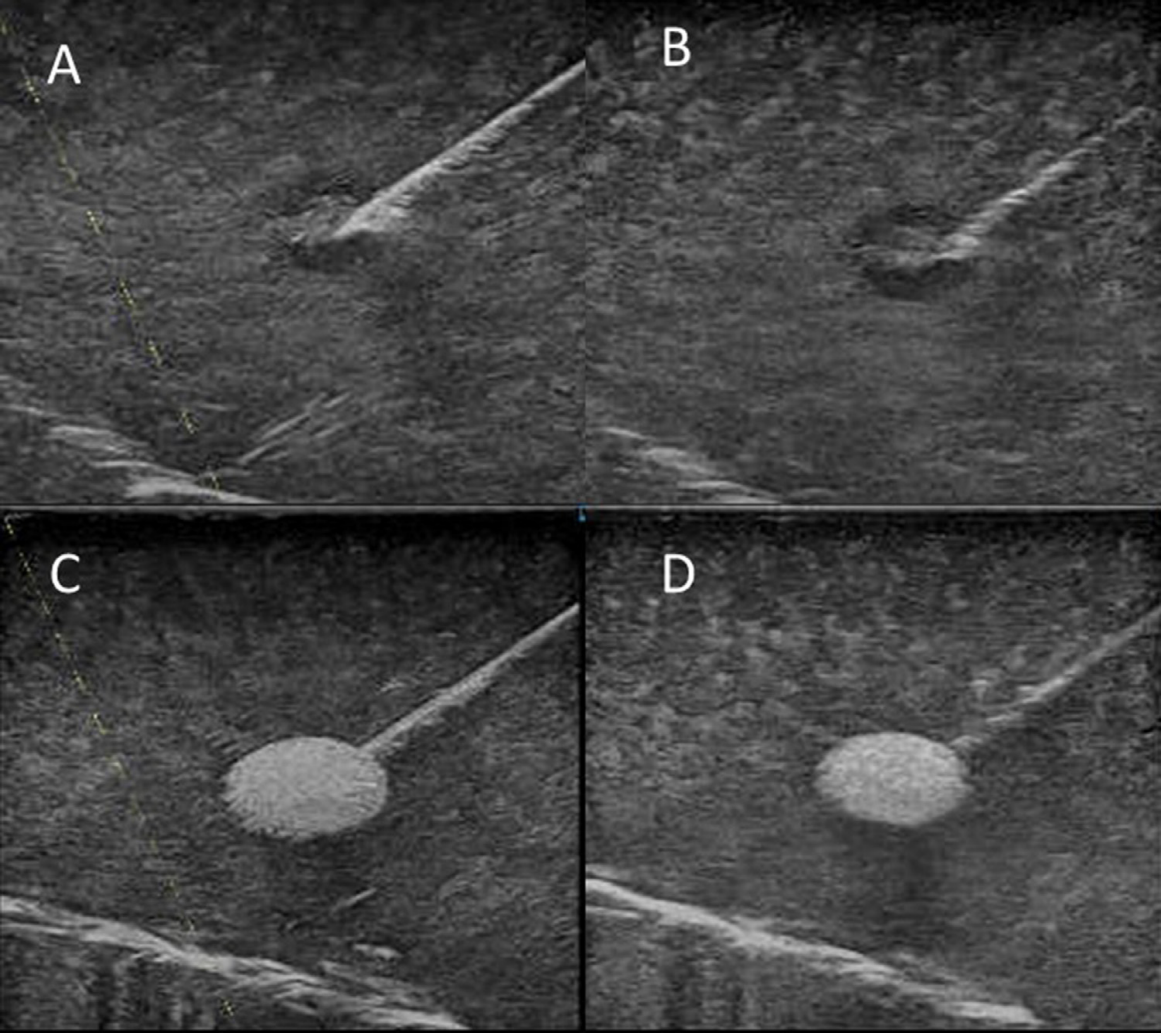
Phantom study Combination of “Survey mode” and “B-Steer+” (A, C) and normal mode (B, D). It can be observed that the needle visibility is higher when the two technologies are used in combination compared with the normal mode.

The visibility of the needle can be improved by using the state-of-the-art technologies “Survey mode” and “B-Steer+” (GE Healthcare, Chicago, IL, USA). In recent years, matrix linear probes, in which multiple elements are arranged in the thickness direction of the probes have been commonly used in routine clinical practice (Fig. 1) [4]. “Survey mode” generates the image and makes the image plane thicker, particularly in the near field, by using all rows of the matrix probe. This thicker near field can sometimes help visualize a needle or the lesion structure in the near field. “B-Steer+” improves the visibility of a needle by increasing the number of times the ultrasound beam is transmitted along a direction perpendicular to the insertion angle of the needle. We have proven in phantom experiments that a combination of these technologies can provide needle visibility. It can be observed that the needle visibility is higher in the combination of “Survey mode” and “B-Steer+” compared with in the normal mode (Fig. 2).

## Case report

**Fig. 3:**
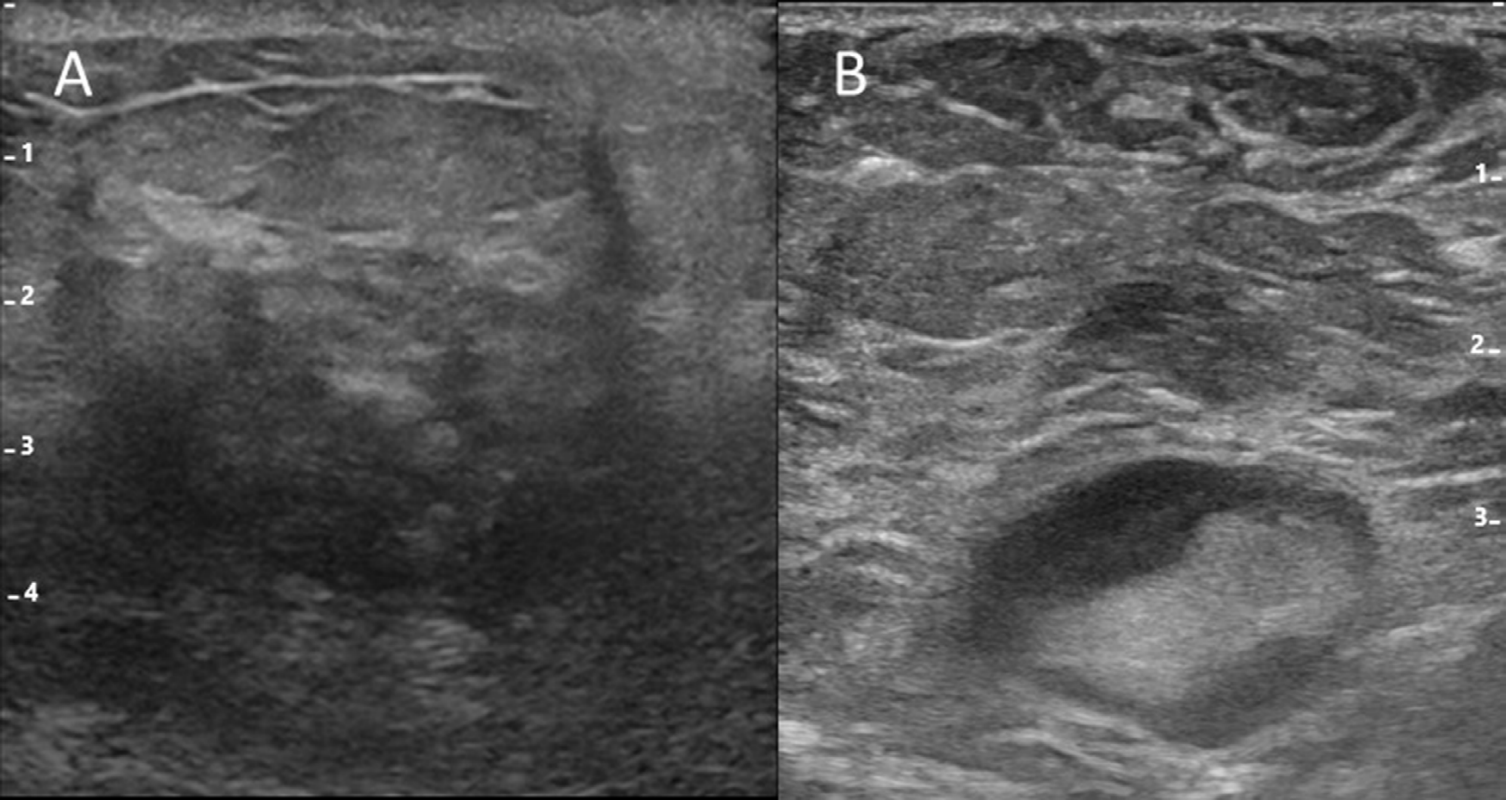
B-mode ultrasound. B-mode ultrasound shows an irregularly shaped mass of 50 mm in the right breast (A). In addition, there were multiple enlarged lymph nodes with irregularly thickened cortices in the right axilla (B).

**Fig. 4:**
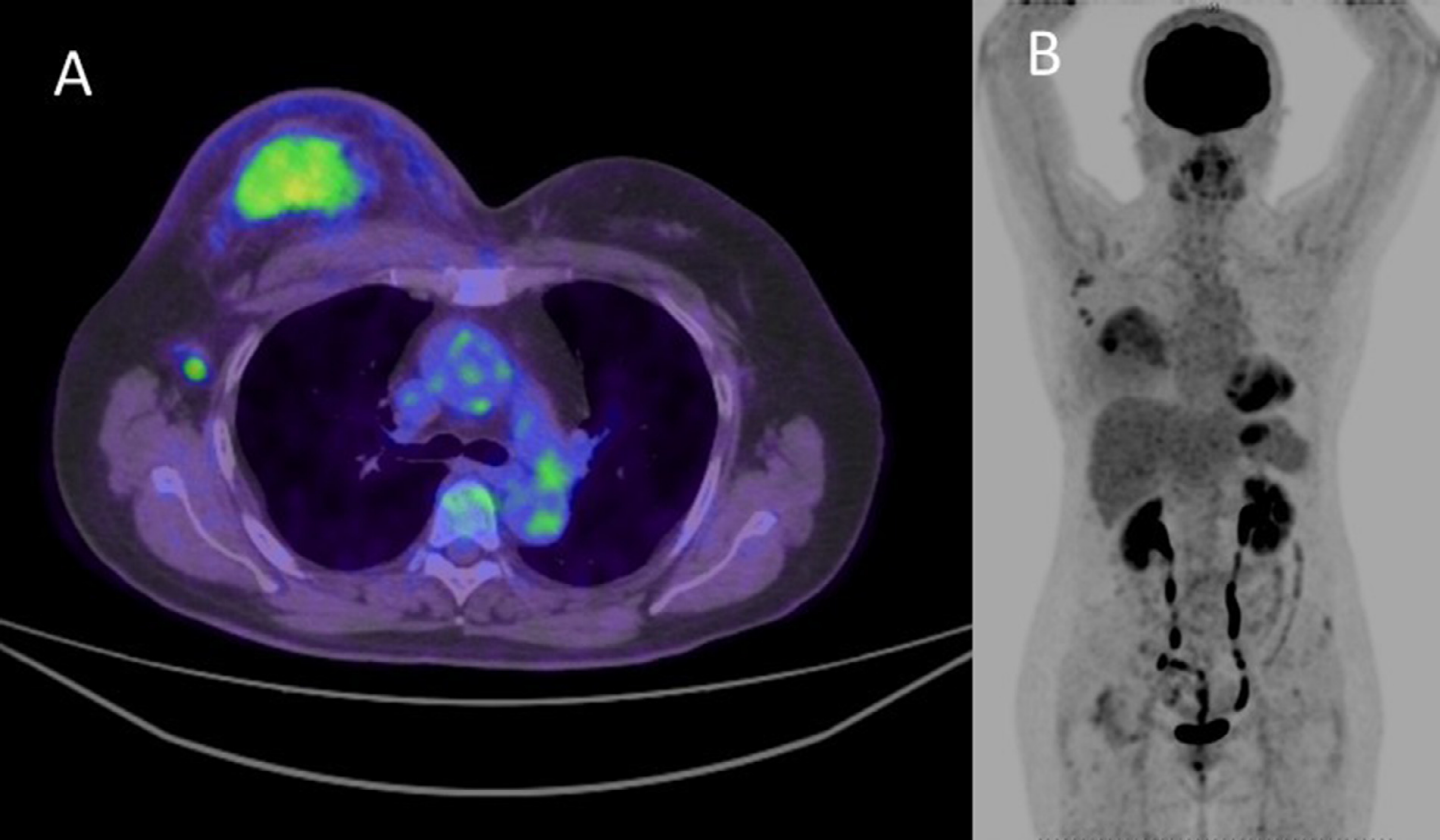
18F-Fluorodeoxyglucose positron-emission tomography/computed tomography outcomes. 18F-Fluorodeoxyglucose positron-emission tomography/computed tomography revealed an intense uptake in the right breast mass (standardized uptake value [SUV]_max_, 5.7) and right axillary lymph nodes (SUV_max_, 3.6) (A). No other areas in which uptakes were noted indicated the presence of suspected distant metastasis (B).

A woman in her 60s with no significant medical history was presented to our hospital with a right breast mass. Ultrasonography was conducted with the use of the latest LOGIQ E10s system with a linear matrix probe ML6-15-D (GE Healthcare, Chicago, IL, USA), and showed an irregularly shaped mass of 50 mm in the right breast. In addition, there were multiple enlarged lymph nodes with irregularly thickened cortex in the right axilla (Fig. 3). 18F-Fluorodeoxyglucose positron-emission tomography/computed tomography revealed intense hot uptake in a right breast mass (standardized uptake value [SUV]_max_, 5.7) and right axillary lymph nodes (SUV_max_, 3.6). No other areas in which uptake was noted indicated suspected distant metastasis (Fig. 4)

**Fig. 5:**
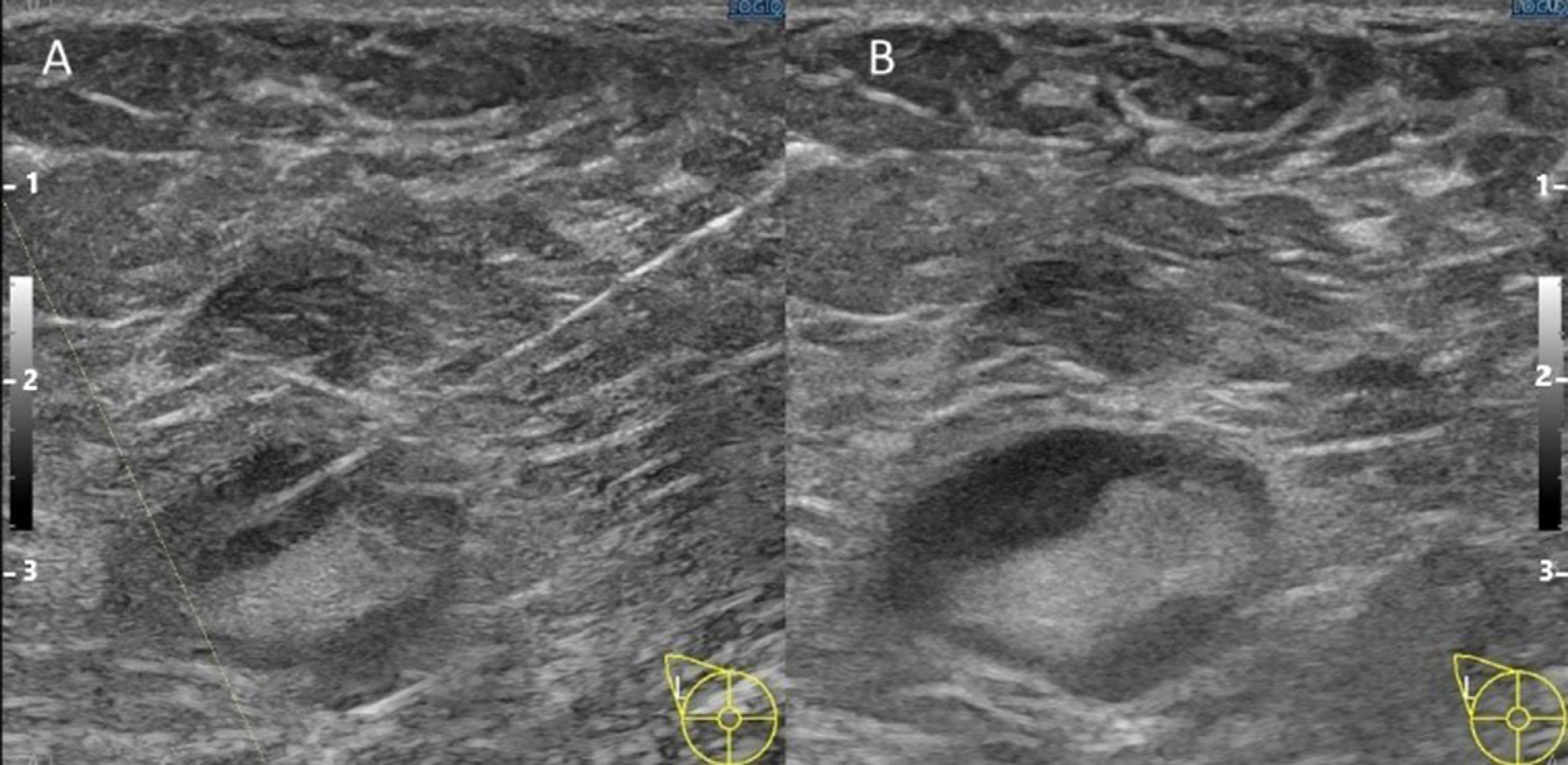
Ultrasound-guided fine-needle (23G) aspiration for left axillary lymph node. Combination of “Survey mode” and “B-Steer+” (A) and normal mode (B). The position of the needle is the same in both images, and the ultrasound mode is switched. The axillary lymph nodes in Figure 5 and Figure 3B are the same. In the normal mode, the visibility of the biopsy needle was poor. However, with the addition of “Survey Mode” and “B-Steer+” the visibility of the needle improved.

We performed a 12G ultrasound-guided aspiration tissue biopsy of the patient's right breast mass. The visibility of the biopsy needle was excellent. A 23G ultrasound-guided fine-needle aspiration was then performed on the left axillary lymph node. In the normal mode, the visibility of the biopsy needle was poor. However, by adding the “Survey mode” to adjust the focal width in the thickness direction of the probe and the “B-Steer+” to electronically steer the direction of the sound wave beam, the visibility of the needle was increased, and the procedure could thus be performed safely and reliably (Fig. 5). The patient was diagnosed with left breast cancer (lobular carcinoma) with left axillary lymph node metastasis, and subsequently underwent chemotherapy.

## Discussion

Breast cancer is the most common cancer affecting women, and its prevalence has been increasing in recent years [5]. Breast cancer frequently metastasizes to the axillary lymph nodes. The diagnosis of axillary lymph node metastasis is very important because it is associated with treatment decisions and patient prognosis. US-guided FNAC is an inexpensive and safe procedure for the diagnosis of axillary lymph nodes. In a study of 1353 patients with breast cancer, the sensitivity and specificity of US-guided FNAC for axillary nodal metastases were 88% and 100%, respectively [6]. Although US-guided FNAC is useful in axillary lymph node diagnosis, it is not easy to perform owing to poor needle visibility in the image, especially when thin needles are used in deep-tissue locations [2,3]. One of the causes is that the needle is so thin such that the ultrasound reflection is weak and needs to be perfectly aligned with the direction of the probe. Another reason is that the needle acts as a perfect US reflector, so angulation of the needle disperses the reflections away from the probe. Using state-of-the-art needle strengthening technology solves these problems and improves needle visibility. Another solution used to improve the visibility of the needle is the case at which the inspector intentionally jiggles the needle to detect the motion in the noisy image.

In conclusion, state-of-the-art enhancement techniques using matrix linear probes may provide better visibility and improve the certainty and efficiency of the examination.

## Patient consent statement

The authors obtained written informed consent from the patient for submission of this manuscript for publication.

## Data availability

Data are available for distribution following reasonable request.

## Author contributions

All authors contributed to the study conception and design. All authors read and approved the final manuscript.

## Ethics approval

All procedures performed in this study that involved human participants were in accordance with the ethical standards of the institutional and/or national research committee, and with the 1964 Declaration of Helsinki and its later amendments, or with comparable ethical standards.
